# Cell-type-specific role of CHK2 in mediating DNA damage-induced G2 cell cycle arrest

**DOI:** 10.1038/s41389-020-0219-y

**Published:** 2020-03-13

**Authors:** Marijn T. M. van Jaarsveld, Difan Deng, Diana Ordoñez-Rueda, Malte Paulsen, Erik A. C. Wiemer, Zhike Zi

**Affiliations:** 1 Max Planck Institute for Molecular Genetics, Otto Warburg Laboratory, Ihnestr. 63-73, 14195 Berlin, Germany; 2 Flow Cytometry Core Facility, European Molecular Biology Laboratory, 69117 Heidelberg, Germany; 3 Department of Medical Oncology, Erasmus MC Cancer Institute, Erasmus University Medical Center, Wytemaweg 80, 3015 CN Rotterdam, The Netherlands; 4 Department of Experimental Toxicology and ZEBET, German Federal Institute for Risk Assessment, Max-Dohrn-Str. 8-10, 10589 Berlin, Germany

**Keywords:** Cancer genetics, Checkpoint signalling

## Abstract

Cancer is a life-threatening disease that affects one in three people. Although most cases are sporadic, cancer risk can be increased by genetic factors. It remains unknown why certain genes predispose for specific forms of cancer only, such as checkpoint protein 2 (*CHK2*), in which gene mutations convey up to twofold higher risk for breast cancer but do not increase lung cancer risk. We have investigated the role of CHK2 and the related kinase checkpoint protein 1 (CHK1) in cell cycle regulation in primary breast and lung primary epithelial cells. At the molecular level, CHK1 activity was higher in lung cells, whereas CHK2 was more active in breast cells. Inhibition of CHK1 profoundly disrupted the cell cycle profile in both lung and breast cells, whereas breast cells were more sensitive toward inhibition of CHK2. Finally, we provide evidence that breast cells require CHK2 to induce a G2–M cell cycle arrest in response of DNA damage, whereas lung cells can partially compensate for the loss of CHK2. Our results provide an explanation as to why *CHK2* germline mutations predispose for breast cancer but not for lung cancer.

## Introduction

Cancer is a disease that can arise in virtually any tissue. Most cases are the result of mutations that occur by chance. However, germline variants can affect cancer risk too. Many cancer predisposition genes (CPGs) have key roles in DNA repair, cell cycle control, and cell survival pathways, which are necessary to maintain genomic integrity. Surprisingly, despite their role in basic cellular programs, CPGs appear to affect cancer development across tissues differently. For instance, mutations of the DNA repair genes breast cancer protein 1 (*BRCA1*) and 2 (*BRCA2*) strongly predispose for breast and ovarian cancer, whereas germline mutations in the DNA repair genes *MSH2*, *MSH6*, and *MLH1* are associated with hereditary nonpolyposis colon cancer.

Why mutations in DNA repair genes predispose for specific cancer types is an outstanding mystery. Since many breast CPGs are involved in the repair of DNA double-strand breaks (DSBs)^1^, it has been proposed that DSB repair is particularly important in breast cells. For instance, there are indications that estrogen metabolism causes DSBs^2,3^. In line with this, we recently showed that the ataxia-telangiectasia mutated kinase (*ATM*)-checkpoint protein 2 (*CHK2*) pathway, which is activated in response to DNA DSBs, is more active in primary breast than in lung cells^4^. Interestingly, both *ATM* and *CHK2* germline mutations predispose for breast cancer^5,6^.

However, DNA repair proteins may contribute to breast cancer risk in additional ways. For instance, mutations in *BRCA1*, which regulates many processes, confer a 10–20% higher risk than *BRCA2* mutations, which functions exclusively in DSB repair^7,8^. In addition, *BRCA1* mutation carriers develop breast cancer at a younger age than *BRCA2* mutation carriers^9^. Both BRCA1 and CHK2 play roles in cell cycle control^10,11^. Since a dysregulated cell cycle can lead to genetic errors and genomic instability, uncontrolled cell division is one of the hallmarks of cancer^12^. It is therefore possible that mutations in *BRCA1* and *CHK2* contribute to cancer development by deregulation of the cell cycle.

To understand differences in tissue-specific cancer risk, we focused on primary breast and lung cells for two reasons. First, breast and lung cancer are among the most common kinds of cancer, suggesting that they have a high cancer risk^13^. Second, several breast CPGs are known, whereas the genetic component of lung tumorigenesis appears to be very small^14^. We observed that breast and lung cells have a different cell cycle distribution, which is reflected in differential CHK1 and CHK2 activity. We provide evidence that breast cells depend on CHK2 to induce a G2 cell cycle arrest in response to DSBs, whereas lung cells appear to have compensatory mechanisms. These findings may help to explain why CHK2 germline mutations predispose for breast cancer but not for lung cancer.

## Results

### CHK1 and CHK2 regulate the cell cycle in primary breast and lung cells differently

We previously observed that the functionally related CHK1 and CHK2 play tissue-specific roles in the DNA damage response in primary breast and lung cells^4^. Interestingly, CHK1 and CHK2 also play roles in cell cycle regulation: CHK1 is required for checkpoints throughout the cell cycle, whereas CHK2 is mostly active during the G1 phase. We therefore set out to compare the cell cycle profile of breast and lung primary cells.

**Fig. 1:**
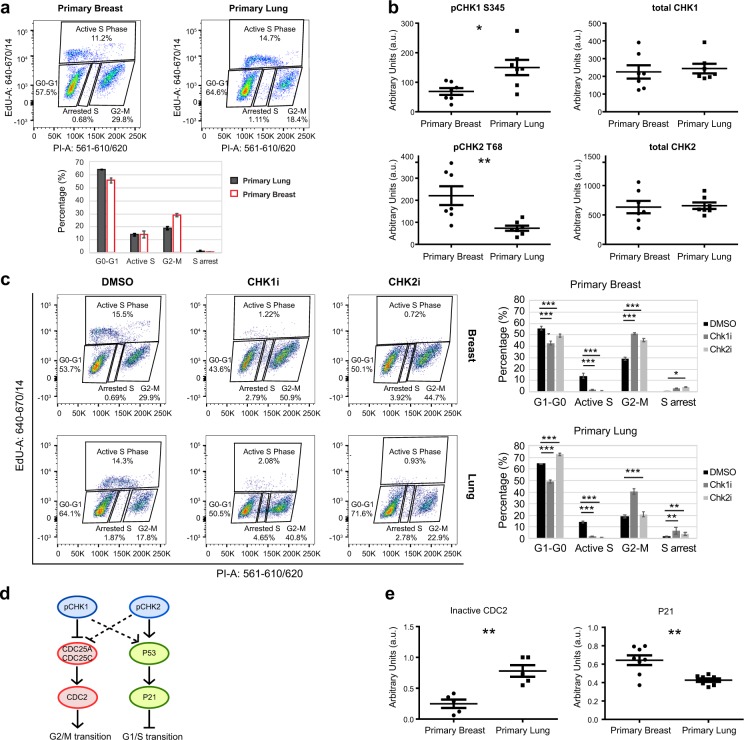
CHK1 and CHK2 dynamics are associated with differential cell cycle regulation in human primary breast and lung cells. **a** Cell cycle profile of human primary breast and lung cells. The results of three independent replicates are depicted (details are available in Supplementary Material). Error bars represent the standard deviation. **b** Expression analysis of total and active CHK1 and CHK2. Lysates from seven primary breast samples and seven primary lung samples, which were isolated from different batches at different times, were analyzed on western blot (Supplementary Fig. 1) and quantified as described in Supplementary Material. A two-sided *t* test was performed to compare the protein levels between primary breast and lung cells. *p < 0.05; **p < 0.01. **c** CHK1 inhibition distorts the cell cycle profile of both lung and breast cells, whereas an effect of CHK2 inhibition is predominantly observed in breast cells. Cells were treated with DMSO, CHK1 (PF477736, Sigma-Aldrich #PZ0186; 1 µM), or CHK2 inhibitor (Cayman Chemicals #17552; 10 µM) for 16 h. During the last 2 h, cells were incubated in the presence of 10 μM EdU. Depicted is a representative experiment (three independent replicates, error bars represent the standard deviation). A two-way ANOVA with Bonferroni post hoc test was performed to compare CHK1- and CHK2-inhibited samples with the DMSO-treated control (**p < 0.01, ***p < 0.001). **d** CHK1 and CHK2 have partially overlapping functions in regulating the cell cycle. Depicted is a cartoon model of regulation of P53, P21, CDC25, and CDC2 by CHK1 and CHK2. CHK1 and CHK2 have overlapping targets, but CHK2 is considered to have a larger role in inducing P53 phosphorylation and P21 activation. CHK1 and CHK2 can both inactivate CDC25C, but CHK1 is considered to be the main inhibitor of CDC25A. CDC25A and CDC25C are phosphatases for CDC2, which remove inhibitory phosphorylation residues, resulting in CDC2 activity and G2-M transition. **e** Differential activity of CHK1 and CHK2 in breast and lung cells is reflected in P21 levels and CDC2 activity. P21 levels are higher in breast cells, whereas inactive CDC2 levels (pCDC2 Y15) are higher in lung cells (details are available in Supplementary Material). A two-sided *t* test was performed to compare the protein levels between breast and lung cells. **p < 0.01.

Both primary breast and lung cells are slowly cycling, with population doubling times of 64 and 42 h approximately^4^. Consistent with the slow population doubling times, the majority of these cells were in G0–G1 phase (breast cells: 55%, lung cells: 65%, Fig. 1a). Remarkably, the fraction of G2–M phase cells appeared to be higher in breast than in lung cells, which may reflect differences in cell cycle regulation.

We next measured total and active CHK1 and CHK2 in breast and lung cells. Whereas total CHK1 and CHK2 had similar expression levels (Fig. 1b, Supplementary Fig. 1), active CHK1 levels were higher in primary lung cells, whereas CHK2 activity seemed to be higher in breast cells.

To investigate if CHK1 and CHK2 play different roles in breast and lung cells, we tested the effect of specific inhibitors on the cell cycle. CHK1 inhibition distorted the cell cycle profile of both cell types, dramatically altering the fraction of cells in G0–G1, S, and G2–M phase (Fig. 1c). CHK2 inhibition led to a loss of active S phase cells in both breast and lung cells. In addition, the percentage of cells in G2–M phase was increased in CHK2-inhibited breast cells, but this fraction was not changed in CHK2-inhibited lung cells. Instead, CHK2-inhibited lung cells displayed a small increase in the fraction of G0–G1 cells. This indicates that CHK2 is required to induce a G1–S phase checkpoint in primary breast cells but not in primary lung cells.

Although CHK1 and CHK2 can both induce cell cycle checkpoints, CHK1 is considered to have a stronger inhibitory effect on the activity of cell division control protein 2 (CDC2)^15^, whereas CHK2 is thought to have a larger role in inducing the expression of the G1–S transition inhibitor P21 (Fig. 1d)^16^. In agreement with an increased CHK1 activity in lung cells, inhibitory phosphorylation of CDC2 appeared to be higher. In contrast, levels of P21 were higher in breast cells, which may reflect increased CHK2 signaling (Fig. 1e).

These results suggest that the differential activity of CHK1 and CHK2 in primary breast and lung cells affects cell cycle regulation.

### DNA damage induces a G2 arrest in CHK2-inhibited lung cells but not in breast cells

CHK1 and CHK2 are known to play key roles in linking DNA damage signaling to cell cycle control. We wondered whether the role of CHK1 and CHK2 in inducing a DNA damage cell cycle checkpoint was different in both cell types. To test this, we made use of the DNA DSB-inducing agent doxorubicin, which is known to induce a G2–M arrest^17^.

**Fig. 2:**
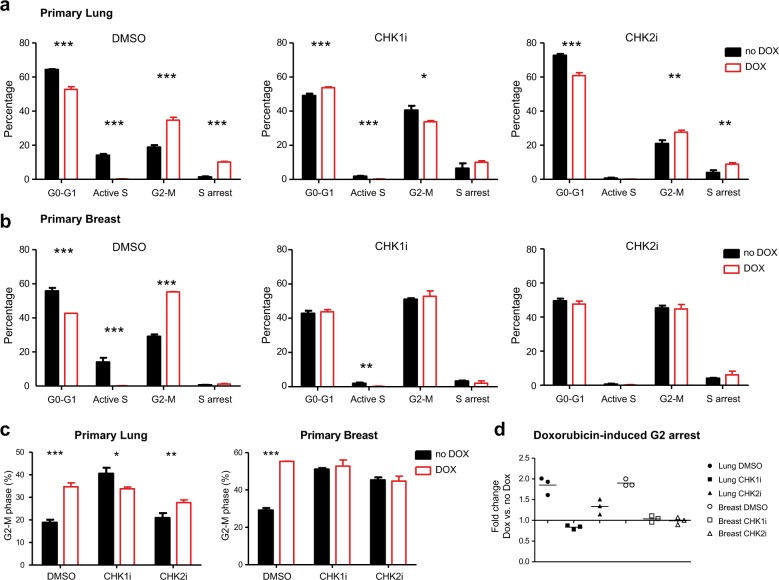
CHK1 and CHK2 play differential roles in DNA damage-induced cell cycle arrests in human primary breast and lung cells. **a**, **b** Effect of doxorubicin treatment in primary lung (**a**) and breast (**b**) cells. Cells were pretreated with DMSO, CHK1 (PF477736; 1 µM), or CHK2 inhibitor (10 µM) for 1 h, followed by control or doxorubicin treatment (0.2 µM for breast, 1 µM for lung) for 16 h. EdU was added 2 h before harvesting cells for EdU detection, PI staining, and FACS analysis. Depicted is a representative experiment (three independent replicates, error bars represent the standard deviation). See also Supplementary Fig. 2a, b. A two-way ANOVA with Bonferroni post hoc test was used to compare the effect of doxorubicin treatment on different cell cycle phases (*p < 0.05, **p < 0.01, ***p < 0.001). **c** Percentage of G2–M cells for DMSO-, CHK1-, or CHK2 inhibitor-treated cells with or without doxorubicin. Depicted is a representative experiment (three independent replicates, error bars represent the standard deviation). A two-way ANOVA with Bonferroni post hoc test was used to compare the effect of doxorubicin treatment on different cell cycle phases (*p < 0.05, **p < 0.01, ***p < 0.001). **d** CHK2 is required for the induction of a doxorubicin-mediated G2–M arrest in breast cells but not in lung cells. Fold change difference in G2–M fraction in doxorubicin-treated cells compared to non-doxorubicin-treated cells.

To measure the effect of DNA damage, primary breast and lung cells were treated with 0.2 or 1 µM doxorubicin, respectively, as these doses give rise to equal intracellular doxorubicin concentrations^4^ and activate CHK1 and CHK2. In both cell types, 16-h exposure to doxorubicin increased the G2–M fraction (Fig. 2a, b, Supplementary Fig. 2a, b). Doxorubicin failed to increase the fraction of G2–M phase cells in CHK1-inhibited breast and lung cells, indicating that CHK1 is required for a doxorubicin-mediated G2–M arrest in these cells (Fig. 2a, b, Supplementary Fig. 2a, b). In contrast, the requirement for CHK2 for a doxorubicin-induced cell cycle arrest appeared to be different for breast and lung cells. Doxorubicin treatment enhanced the G2–M fraction in CHK2-inhibited lung cells but not in CHK2-inhibited breast cells (Fig. 2a–d).

**Fig. 3:**
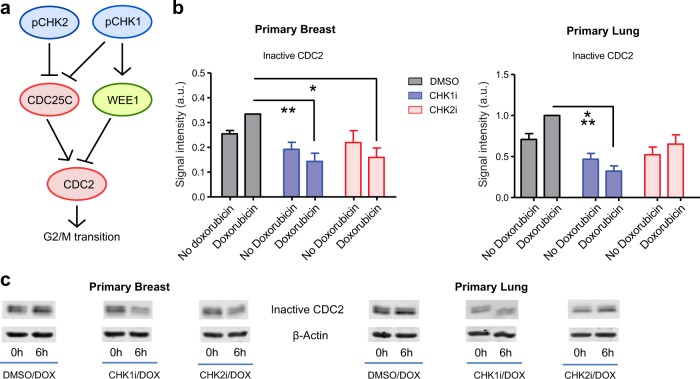
CHK2 signaling is required to inhibit CDC2 in primary breast cells but not in human primary lung cells. **a** Regulation of CDC2 dynamics by CHK1 and CHK2. CDC2, the activity of which is required for G2–M transitions, is kept inactive by phosphorylation on Y15. The kinase Wee1 phosphorylates Y15, whereas CDC25C dephosphorylates it. CHK1 and CHK2 inhibit CDC25C, and CHK1 activates Wee1. **b** Inhibitory phosphorylation of CDC2 (pCDC2 Y15) in human breast and lung cells before and 6 h after doxorubicin treatment (0.2 µM for breast, 1 µM for lung). Cells were pretreated for 1 h with DMSO, CHK1 inhibitor (1 µM), or CHK2 inhibitor (10 µM). Depicted is the average level of inactive CDC2 (pCDC2 Y15) expression (*n* = 3). Error bars represent the SEM. A one-way ANOVA with Bonferroni post hoc test was used to assess whether differences between (1) inhibitor treatment vs. DMSO and (2) doxorubicin treatment vs. no doxorubicin were statistically significant (*p < 0.05, **p < 0.01, ***p < 0.001). **c** Inhibitory phosphorylation of CDC2 (pCDC2 Y15) in human breast and lung cells with 6 h of doxorubicin treatment (0.2 µM for breast, 1 µM for lung). Cells were pretreated for 1 h with DMSO, CHK1 inhibitor (1 µM), or CHK2 inhibitor (10 µM). Depicted is a representative blot (*n* = 3).

To understand why CHK2 is required for G2–M arrest in breast but not in lung cells, we focused on its downstream effector CDC2, which in complex together with Cyclin B1 constitutes the master regulator of the G2–M transition (Fig. 3a). Doxorubicin treatment increased CDC2 inhibitory phosphorylation (pCDC2 Y15) at 6 h (Fig. 3b, c), coinciding with peak levels of active CHK1 and CHK2 (Supplementary Fig. 3a). This is consistent with the induction of a G2–M arrest. Afterwards, a loss of Cyclin B1 presumably sustained cell cycle arrest (Supplementary Fig. 3a–c)^18^. Next, we examined whether doxorubicin treatment in CHK1- and CHK2-inhibited cells was able to increase pCDC2 phosphorylation. In CHK1-inhibited cells, doxorubicin treatment failed to augment inactive CDC2 levels (Fig. 3b, c, Supplementary Fig. 3a). This indicates that CHK1 is required for the inhibition of CDC2 and may explain why doxorubicin does not enhance the fraction of cells in G2–M phase after CHK1 inhibition (Fig. 2a). Interestingly, we observed that the effect of doxorubicin on CHK2-inhibited cells was tissue-specific. In CHK2-inhibited lung cells, doxorubicin induced CDC2 inactivation (Fig. 3b, c), which may reflect the induction of a G2–M cell cycle arrest. However, in CHK2-inhibited breast cells, doxorubicin did not elevate CDC2 inhibitory phosphorylation, which may explain why the G2–M cell fraction did not increase after doxorubicin exposure (Fig. 2b–d).

Together, these findings indicate that CHK2 has a tissue-specific role in mediating DNA damage induced G2-M arrest.

## Discussion

In this exploratory study, we present evidence that CHK1 and CHK2 play tissue-specific roles in cell cycle regulation. CHK1 activity is higher in lung cells and CHK1 inhibition has a more profound effect on cell cycle regulation in lung cells than in breast cells. In contrast, active CHK2 levels are higher in breast cells and CHK2 inhibition distorts the cell cycle profile of breast cells, whereas the effect on lung cells is minor.

CHK1 and CHK2 are both activated upon DNA damage and can regulate cell division. CHK1 activity has been mostly implicated in the intra-S phase cell cycle checkpoint and the G2–M transition^19^. In contrast, CHK2 activity is associated with G1–S and G2–M arrests. CHK1 and CHK2 regulate different cell cycle proteins. An important group of cell cycle regulators is the CDC25 family, which are phosphatases that activate CDC2 and/or cyclin-dependent kinase 2. Both CHK1 and CHK2 can inhibit CDC25C^20,21^, whereas CHK1 is more effective in phosphorylating CDC25A^15^ and CDC25B^22^. Through inhibition of the CDC25 family, CHK1 and CHK2 may prevent CDC2 activation. In addition, CHK1 can contribute to inactive CDC2 phosphorylation by stimulating the kinase Wee1^23^. Since CHK1 activity is higher in lung cells, this may explain why the levels of inactive CDC2 are higher in lung cells. Another mechanism by which CHK2 and CHK1 regulate the cell cycle is through phosphorylation of P53 and upregulation of P21^24,25^. In line with the notion that CHK2 has a larger role than CHK1 in inducing P21 expression^16^, P21 levels are higher in breast cells.

Interestingly, we previously observed that CHK1 is preferentially upregulated after DNA damage in lung cells, whereas CHK2 activation was stronger in breast cells after DNA DSBs^4^. We therefore tested the role of CHK1 and/or CHK2 in DNA damage-induced cell cycle arrests. When breast and lung cells were treated with doxorubicin, a twofold increase in the fraction of G2–M phase cells was observed. This increase could not be observed in CHK1-inhibited cells, in line with the notion that CHK1 activity is essential for the induction of a G2–M arrest^26^. Remarkably, doxorubicin treatment enhanced the G2–M phase fraction of CHK2-inhibited lung cells but not in CHK2-inhibited breast cells. An explanation for this may be the observation that CHK2-inhibited lung cells were still able to inactivate CDC2, whereas CHK2-inhibited breast cells were not. The peak CDC2 inhibitory phosphorylation occurred at 6 h, coinciding with the pCHK2 and pCHK1 activity peaks. Interestingly, afterwards pCDC2 levels appeared to decrease, as has been described to be a cell-type-specific phenomenon^27,28^ (reviewed in ref. ^18^). Since the CDC2/Cyclin B complex is a better substrate for tyrosine kinase Wee1^29^ than CDC2 alone, we hypothesize that the drop in Cyclin B levels may be responsible for this decrease. Thus the initial inactivation of CDC2 triggers an early G2–M arrest, whereas the drop in Cyclin B1 levels at a later stage may maintain G2–M phase arrest. The mechanism responsible for the loss of Cyclin B1 may be the upregulation of P53^18^.

Cell cycle checkpoints are important to prevent transmission of damaged DNA to the daughter cells, and hence CHK1 and CHK2 can protect cells against cancer^30^. Concurringly, sporadic mutations of *CHK1* and *CHK2* have been found in most types of cancer. In addition, germline mutations of CHK2 appear to predispose for certain types of cancer. People who harbor truncating CHK2 mutations (e.g., CHEK2*1100delC mutation) have an approximately twofold increased risk of developing breast cancer^31,32^. Carriers also have an increased likelihood to develop prostate^33,34^ and colon cancer^35–37^, but, intriguingly, no increased risk of lung cancer^38,39^.

Considering the pivotal importance of cell cycle arrests to prevent genomic instability, our data may provide an explanation for why loss of *CHK2* predisposes for breast cancer but not for lung cancer. Since mice harboring a CHK2*1100delC mutation did not show the tissue-specific bias observed in humans^40^, a better disease model is needed to understand *CHK2*-mediated cancer predisposition. The comparative analysis of healthy primary epithelial cells may provide further insights into the relation between loss of *CHK2* and tissue-specific cancer development.

## Supplementary information

Supplementary Information
