# Supplementary material for: Cell-type-specific role of CHK2 in mediating DNA damage-induced G2 cell cycle arrest
Source: Oncogenesis. 2020 Mar 13;9(3):35. doi: 10.1038/s41389-020-0219-y (PMC7070093; doi:10.1038/s41389-020-0219-y)
Supplement: Supplementary file 1 — Supplementary Information [file 41389_2020_219_MOESM1_ESM.pdf]

Supplementary Information

Cell type-specific role of CHK2 in mediating DNA damage-induced G2 cell cycle arrest

Marijn T.M. van Jaarsveld[1], Difan Deng[1], Diana Ordoñez-Rueda[2], Malte Paulsen[2], Erik A.C. Wiemer[3], Zhike Zi[1,4 †]

[1] Max Planck Institute for Molecular Genetics, Otto Warburg Laboratory, Ihnestr. 63-73, 14195 Berlin, Germany

[2] Flow Cytometry Core Facility, European Molecular Biology Laboratory, 69117 Heidelberg, Germany

[3] Department of Medical Oncology, Erasmus MC Cancer Institute, Erasmus University Medical Center, Wytemaweg 80, 3015 CN Rotterdam, The Netherlands

[4] German Federal Institute for Risk Assessment, Department Experimental Toxicology and ZEBET, Max-Dohrn-Str. 8-10, 10589 Berlin, Germany

[†] Corresponding author. Email: zhike.zi@molgen.mpg.de

Supplementary Figures

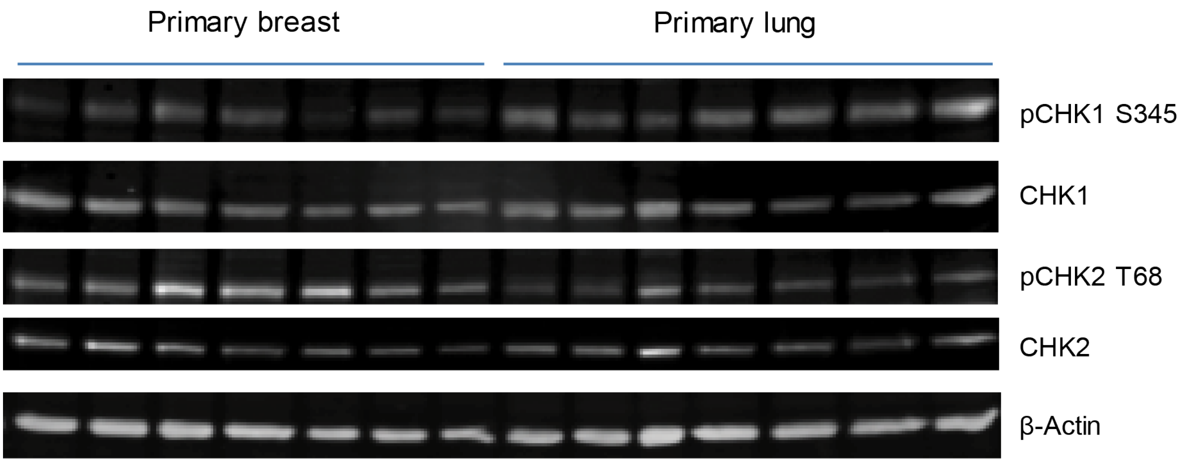

Figure S1: Expression analysis of total and active CHK1 and CHK2 in human primary breast and lung cells

Expression analysis of total and active CHK1 and CHK2. Depicted are results of western blot analysis of 7 primary breast cell samples and 7 primary lung cell samples, which were isolated from different primary cell culture batches at different times. From each sample, a protein lysate equivalent to 93.000 cells was loaded.

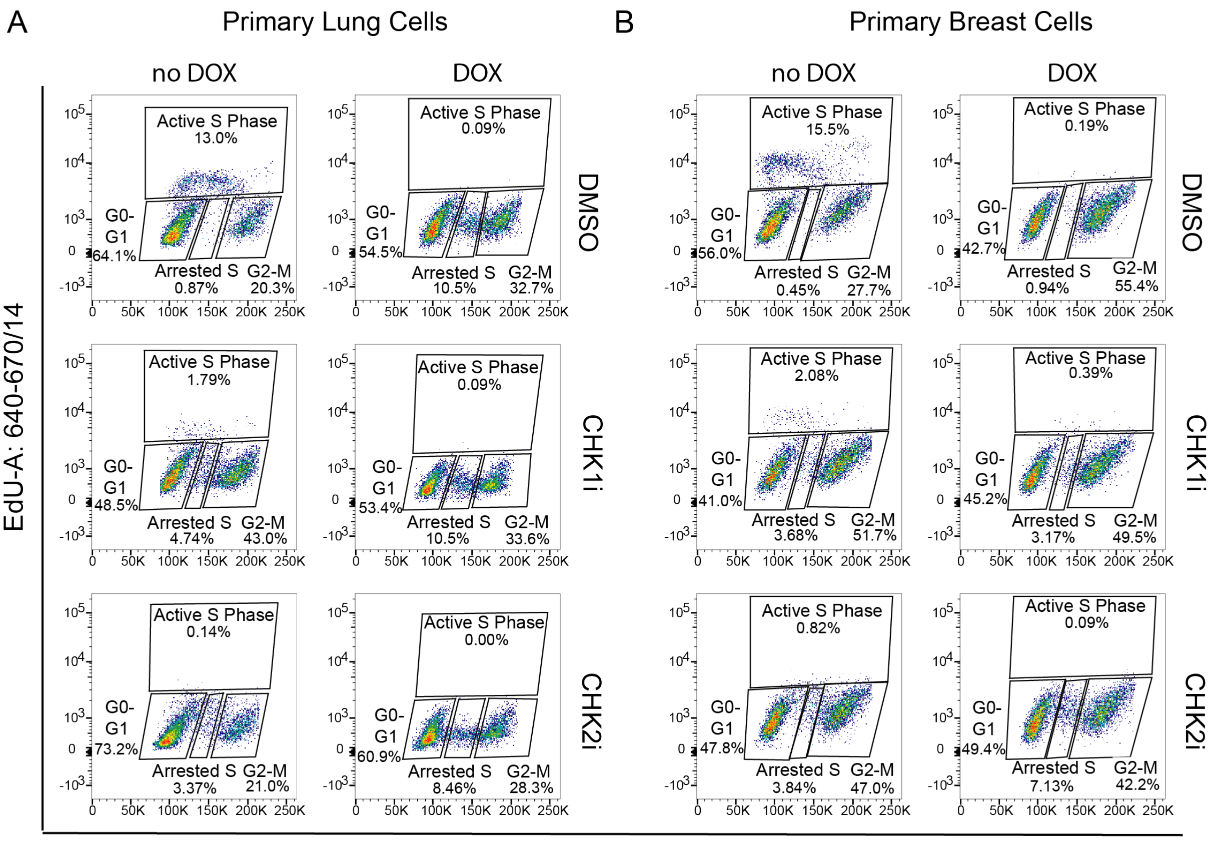

Figure S2: Role of CHK1 and CHK2 in DNA-damage induced cell cycle checkpoints

A and **B**: Effect of doxorubicin treatment in primary lung (**A**) and breast (**B**) cells. Cells were pretreated with DMSO, CHK1 (PF477736; 1 µM) or CHK2 inhibitor (10 µM) for 1 hour, followed by control or doxorubicin treatment (0.2 µM for breast, 1 µM for lung) for 16 hours. EdU was added 2 hours before harvesting cells for EdU detection, PI staining and FACS analysis (Details are in the Supplemental Material). Depicted is a representative experiment.

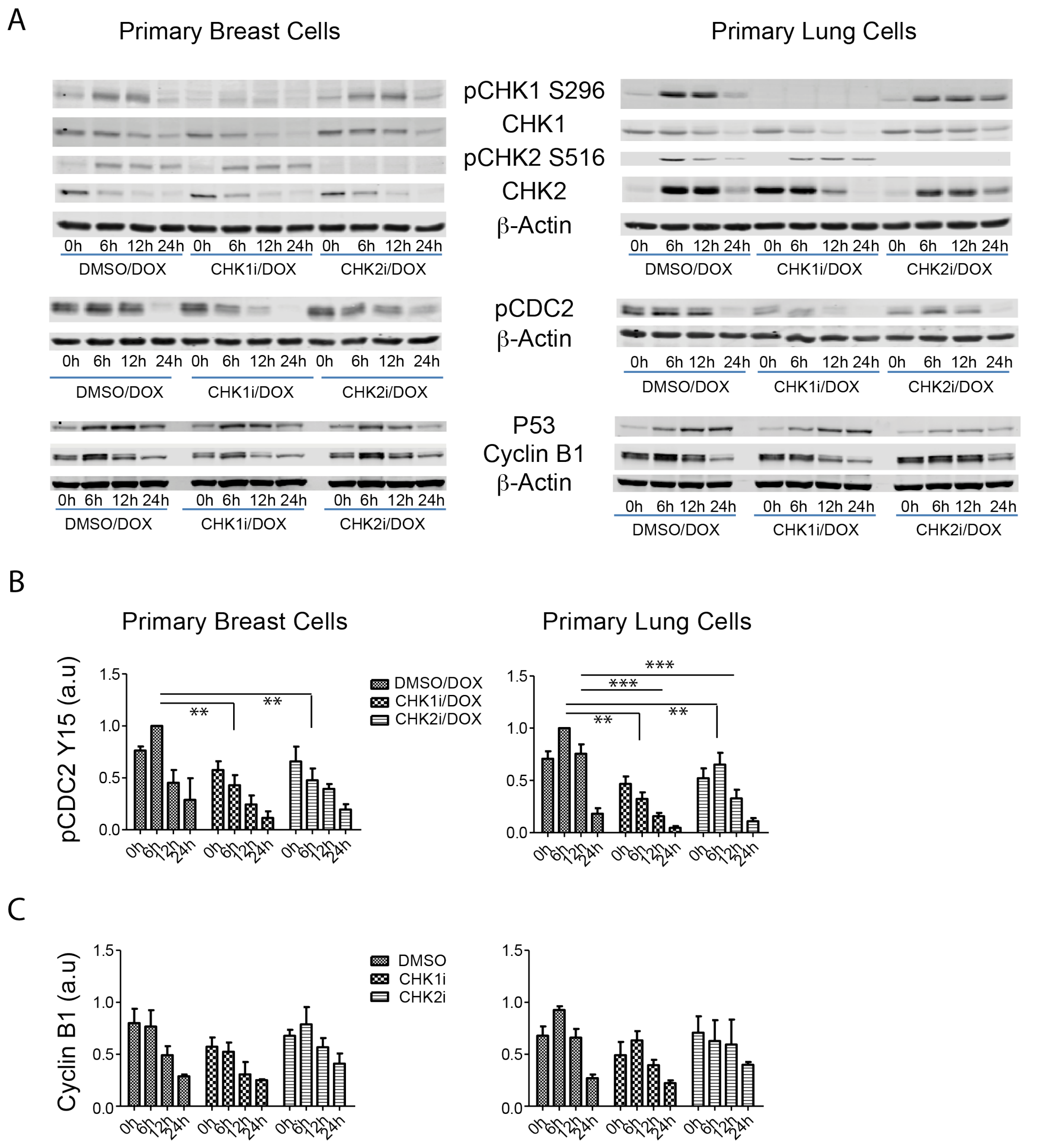

Figure S3: Regulation of the Cyclin B and CDC2 in human primary breast and lung cells

A: Expression analysis of total and active CHK1 and CHK2, pCDC2 Y15, p53 and Cyclin B1. Depicted are results of a representative western blot analysis of primary breast samples and primary lung samples (n=3-5).

B-C: Dynamics of pCDC2 Y15 and Cyclin B1 after doxorubicin treatment. Cells were pretreated for 1 hour with DMSO, CHK1 inhibitor (1 μM) or CHK2 inhibitor (10 μM). Depicted is the average level of inactive CDC2 (pCDC2 Y15) and Cyclin B1 expression (n=3). Error bars represent the SEM. A one-way ANOVA with Bonferroni post-hoc test was used to assess if differences between inhibitor treatment vs. DMSO were statistically significant (**=p<0.01, ***=p<0.001).

Supplemental Materials and Methods

Cell culture and reagents

Primary human mammary epithelial cells (HMEpC; lot number 1462342) and human small airway epithelial cells (HSAEpC; lot number NRG1000141) were obtained from Invitrogen and Merck, respectively. Primary breast cells were grown in Mammary Epithelial Cell Growth Medium from Promocell and Primary lung cells in BronchiaLife B/T medium from Cell Systems. We selected cells from these donors, after extensive characterization of the DNA damage response and population doubling time proved them to be comparable to primary cells of other donors (see reference [1]). These cells are known to retain their characteristics for at least 16 population doublings. Upon arrival, the cryovials were thawed and grown to population doubling 5, after which they were frozen and aliquotted. For every experiment, a cryovial was thawed and grown until population doubling 10-12, at which point experiments were performed. The population doubling time of primary breast cells was 64 hours and the population doubling time of primary lung cells was 42 hours. Before experiments cells were equally distributed in cell culture plates or dishes. The dishes/wells were randomly assigned to treatments and the samples were processed and analyzed in a blinded fashion. The patient-formulated doxorubicin solution (2 mg/mL) was obtained from a local pharmacy. CHK1 inhibitor PF477736 (PZ-0186) was acquired from Sigma-Aldrich and CHK2 inhibitor II (17552) was obtained from Cayman Chemicals.

Quantitative western blot

Cell lysates were prepared using RIPA buffer from Cell Signaling Technology (CST, Catalog #9806, 20 mM Tris-HCl pH 7.5, 150 mM NaCl, 1 mM Na2EDTA, 1 mM EGTA, 1% NP-40, 1% sodium deoxycholate, 2.5 mM sodium pyrophosphate, 1 mM β-glycerophosphate, 1 mM Na3VO4, 1 µg/mL leupeptin), supplemented with 1 mM PMSF, 5 mM NaF, protease inhibitor cocktail (Roche, Catalog #04 693 132 001) and phosphatase inhibitor cocktail (Roche, Catalog #04 906 845 001). 20-30 µg of

protein samples (determined by BCA assay) were used for western blot experiments. Afterwards, blots were incubated with Odyssey blocking buffer for 1 hour, followed by primary antibody incubation at 4 ^{0}C overnight. The antibodies for CHK1 (pan-specific; CST, #2360), pCHK1 S345 (CST, #2348), pCHK1 S296 (CST, #2349), CHK2 (pan-specific; CST, #6334), pCHK2 T68 (CST, #2197), pCHK2 S516 (CST, #2669), p21 (CST, #2947), pCDC2 Y15 (CST, #4539), Cyclin B1 (CST, #12231) and b-Actin (CST, #3700) were used at a 1:1000 concentration in 5% BSA/TBST. P53 antibody from Santa Cruz (SC-126) was used 1:200 in 5% Milk/TBST. Goat-anti rabbit Dylight 800 (CST, #5151) and Goat-anti rabbit Dylight 680 (CST, #5470) were used as secondary antibodies. Signal was detected using the LI-COR Odyssey CLx scanner. Blots were incubated with different antibodies on subsequent days.

FACS analysis

Cells were incubated with EdU (10 μM final concentration) for 2 hours before harvest. EdU Pulsed cells were fixed with absolute ethanol 70% (Sigma-Aldrich) and kept at -20°C until analysis. The FACS samples were blinded and independently analyzed in the Flow Cytometry Core Facility at EMBL. Incorporated EdU was detected using the Click-iT™ Plus EdU Cell Proliferation Kit (Thermo Fisher Scientific, C10640) following the indications of the manufacturer. In order to determine DNA content, cells were stained with Propidium Iodide (PI) in a solution containing 0.1% Triton X-100 and RNase at 200 μg/ml. Cells were incubated 15 min at 37°C protected from light. Stained samples were analyzed immediately after labeling on a LSRFortessa™ (BD-Biosciences). PI signal was acquired by the 561nm laser line and a 610/20 bandpass filter. The Alexa Fluor® picolyl azide (incorporated EdU) was acquired using a 670/14 bandpass filter and a 640nm laser line. Doublets were carefully excluded by plotting PI-area versus PI-width. Post-acquisition analysis was done in FlowJo software (BD Biosciences).

Statistical Analysis

All experiments were conducted at least three times. This sample size was sufficient to detect a 25% difference between populations with a standard deviation of ~10% of the mean (determined by earlier studies) with 80% power, alpha 5%. For Fig. 1b and 1e, an independent sample 2-sided t-test was performed. For Fig. 1c, Fig. 2a-c, a two-way ANOVA with Bonferroni post hoc test was used. A one-way ANOVA with Bonferroni post-hoc tests was used to compare the columns in Fig. 3b and Supplementary Fig. 3b. Asterisks indicate significant differences.

Supplemental References

1 van Jaarsveld MT, Deng, D., Wiemer, E.A., Zi, Z. Tissue-specific Chk1 activation determines apoptosis by regulating the balance of p53 and p21. *iScience* 2019; **12**: 27-40.
